# Autologous Splenocyte Reinfusion Improves Antibody-Mediated Immune Response to the 23-Valent Pneumococcal Polysaccharide-Based Vaccine in Splenectomized Mice

**DOI:** 10.3390/biom10050704

**Published:** 2020-05-01

**Authors:** Shengwen Calvin Li, Mustafa H. Kabeer

**Affiliations:** 1 Neuro-Oncology and Stem Cell Research Laboratory (NSCL), Center for Neuroscience Research (CNR), CHOC Children’s Research Institute (CCRI), Children’s Hospital of Orange County (CHOC), 1201 West La Veta Ave., Orange, CA 92868-3874, USA; 2 Department of Neurology, University of California-Irvine School of Medicine, 200 S Manchester Ave Ste 206, Orange, CA 92868, USA; 3 Division of Pediatric General and Thoracic Surgery, CHOC Children’s Hospital, 1201 West La Veta Ave., Orange, CA 92868, USA; mkabeer@choc.org; 4 Department of Surgery, University of California-Irvine School of Medicine, 333 City Blvd. West, Suite 700, Orange, CA 92868, USA

**Keywords:** reinfusion of autologous splenocytes, antibody response to PNEUMOVAX^®^23 vaccines, splenectomy

## Abstract

Common clinical options, currently, for necessary splenectomy are vaccinations and antibiotic prophylaxis. However, despite these two adjuncts, there still occur numerous cases of overwhelming post-splenectomy infection. To examine whether reperfusion of critical splenic lymphocytes could boost immune response, we harvested splenic lymphocytes, reperfused the autologous lymphocytes, and then administered a pneumococcal vaccine (PNEUMOVAX^®^23, i.e., PPSV23) in splenectomized mice. We found that splenectomy impaired the immune response in the splenectomized group compared to the non-splenectomized group; the splenectomized group with lymphocyte reinfusion had a higher response to polysaccharide vaccination based on antibody titer than the splenectomized group without lymphocyte reinfusion. The sham group with the native spleen had the most elevated antibody titer against the PPSV23 polysaccharide antigen. This may be additive, resulting from contributions of the splenic structure, along with the phagocytic function of the spleen and its constituent cells affecting the antibody response. Reinfusion of splenic lymphocytes may enhance immunity without the complications associated with splenic fragment autotransplantation, which never gained acceptance. This technique is safe and simple since the splenic lymphocytes are autologous and, therefore, not self-reactive, and very similar to autologous blood transfusion. This concept may be beneficial in cases of unavoidable splenectomy, especially in pediatric cases.

## 1. Introduction

Current clinical practices in pediatric surgery maintain high importance on splenic preservation due to concerns of future infectious risks from gram-negative encapsulated microorganisms. Significant efforts and focus are placed on maintaining splenic integrity in pediatric patients. In cases of traumatic injury, patients are placed on bedrest with sequential hemoglobin monitoring, along with close observation of vital signs (especially heart rate) in order to avoid performing a splenectomy. However, there are groups of patients with hemoglobinopathies and blood dyscrasias, such as sickle cell anemia, thalassemia, and hereditary spherocytosis, with resulting splenomegaly from blood sequestration resulting in transfusion requirements, eventually necessitating splenectomy. Often, total splenectomy is performed, but more attempts are now made to perform a partial splenectomy to allow for resolution or diminishing the effects of blood sequestration and destruction, but still maintain immune function, as well as avoid other complications of total splenectomy, such as pulmonary hypertension, which can develop over time [1]. Worldwide, other medical conditions, such as malaria, can lead to a significantly higher incidence of splenomegaly and rupture, requiring operative intervention in a setting where immune prophylaxis with vaccination and medication may be either unavailable or difficult to obtain [2]. Splenectomized patients, especially children, face increased risk of overwhelming post-splenectomy infection (OPSI) due to sepsis from gram-negative encapsulated microorganisms. The current wisdom in surgical treatment has been to conserve the spleen whenever possible, especially in pediatric patients, which account for 85% of such cases [3]. Spleen conservation has been proven as most effective for long-term immune protection, and can also be further boosted by immunization, with polyvalent pneumococcal polysaccharide, in patients with sickle cell anemia [4].

The structure of the spleen is an integral part of its immune function. The vascular flow through the sinusoids allows antigenic debris to be presented to the resident reticuloendothelial cells lining the sinuses. These cells, in turn, can present processed antigen to activate other cells within the immune system. Therefore, it has long been felt that this integrity of the spleen structure must be preserved to maintain function. The spleen also serves as a filter to intravascular bacterial contaminants, a possible way of decreasing the risk of OPSI and other post-splenectomy complications. Structural and functional attributes of the spleen prompted surgeons to save the spleen entirely, if possible, or to salvage a portion of the spleen by performing partial splenectomy, despite significant technological complexity and increased bleeding risk.

In the past, numerous studies have examined the feasibility of autologous splenic transplantation as a viable alternative in unsalvageable cases requiring splenectomy. Such autologous splenic transplantation led to improved antibody responses [5], along with increased levels of opsonins and tuftsin. There also seems to be some suggestion that patients suffering from a traumatic splenic injury, requiring splenectomy, may be at a slightly lower risk of OPSI due to a phenomenon of splenosis, resulting in autotransplantation of splenic tissue due to the impact on the spleen lending itself to some retained immune function. Supporting data for such benefits were seen in a study with 52 patients, including 11 children (four girls, seven boys), who suffered from abdominal trauma and underwent total splenectomy [6]. Certain studies have shown that in order to achieve any benefit in humoral immunity, at least half of the spleen should be retained [5]. Some studies reported beneficial outcomes from splenic autotransplants within the mesentery, in comparison to intramuscular transplants [7].

Additionally, intraperitoneal autotransplantation of splenic tissue improved antibody titers. Further, retention of at least 50% splenic volume increased survival rates when compared to a total splenectomy in a mouse model after a *Streptococcal* challenge [8]. However, patients who undergo splenectomy for clinical indications will not have symptom or disease resolution by preserving such a large portion of the spleen, and usually partial splenectomies aim at preserving 25–30% of splenic mass, along with its feeding vascular pedicle.

Splenic autotransplantation was never adapted in clinical practice due to frequent complications, such as autotransplant fibrosis, aseptic necrosis, or bowel adhesion and obstruction [9]. Such complications often necessitated further surgery with no lasting benefits. Additionally, there is some experimental evidence of a lack of sustained efficacy of such autotransplants, which undergo approximately 8% autonecrosis each year, and soon fall optimal efficacy [10]. Aside from function, it has also been noted that transplanted spleen sections have been found to have decreased the size of the periarteriolar lymphatic sheath (PALS) along with changes in the density of B-cell, macrophages, and T-cell ratios. Not only have changes in parenchymal architecture been observed, but vasculature within the spleen may also be altered by dilation of vessels in the marginal zone, pulp cords, and red pulp, where antigen presentation occurs in the spleen.

As stated above, the risks of OPSI are elevated in all splenectomized patients, and the most frequent causes of OPSI include encapsulated organisms, specifically *Streptococcus pneumoniae*. Currently, vaccine and antibiotic prophylaxis are used to help prevent OPSI in splenectomized patients. Although polyvalent pneumococcal vaccines are available and used for prophylaxis in cases of necessary splenectomy, there are cases where patients have still succumbed to fatal sepsis due to OPSI. Numerous studies also demonstrate that there exist significant lapses in adhering to recommendations for vaccination prior to splenectomy as well as to prophylactic antibiotics, as indicated, leaving patients at risk. Finally, the problems are compounded when patients globally with this condition potentially face problems with access to either medical care or to vaccines and antibiotics. Even when patients get vaccinations, some studies have shown that serum titers against specific pneumococcal subtypes decline over time to non-protective levels. Indeed, although vaccination is most effective if given before splenectomy, this is not always possible.

*Streptococcus pneumoniae* is a causative agent of community-acquired pneumonia, leading the 2012 Advisory Committee on Immunization Practices recommend the vaccination with Prevnar^®^13 (protein-polysaccharide conjugate vaccine with 13 serotypes; PCV13), followed by Pneumovax^®^ 23 (polysaccharide-based vaccine with 23 serotypes; PPSV23) [10,11]. There are cases where patients have still succumbed to fatal sepsis due to the failure of pneumococcal vaccine in children with the sickle-cell disease. This is due to two immunological defects, functional asplenia, and decreased pneumococcal serum opsonins [12], or fatal pneumococcal bacteremia in a vaccinated splenectomized child [13], or occurrence after vaccination against pneumococcal pneumonia subtypes in an asplenic patient [14].

Mice that received type III pneumococcal capsular polysaccharide vaccine 1 μg IP, 48 h post-splenectomy, and 7 days before the challenge with aerosolized type III *Streptococcus pneumoniae* had significantly higher mortality (96%) compared to immunized controls (64%) (*p* < 0.002) [15]. The serum titers against specific pneumococcal subtypes decline over time to non-protective levels in children post-splenectomy [16]. Vaccination is most effective if given before splenectomy [15]. Due to the inability to pre-vaccinate for emergent splenectomies secondary to trauma, the inefficacy of splenic autotransplants, and the potential benefits of retaining immunocompetent autologous splenic cells alongside all of the recommended adjuncts of vaccination and antibiotic prophylaxis, we advocate for researching and implementing autologous splenic lymphocyte reinfusions post-splenectomy. In this study, we evaluate our method by comparing antigen challenges to mice with and without autologous splenocyte reinfusion compared to sham non-splenectomized mice (sham/control).

Although we have a better understanding of the complexity of the human immune system and its cellular interactions and signals of response to antigen challenge, many areas yield contradictory and sometimes confusing data. In the area of B cell humoral response, it is unclear exactly how much T cell interaction is either necessary or detected in eliciting the full gamut of antibody response to T-independent antigens from polysaccharide vaccines. This study was designed to begin to address the question of whether or not there may be any health benefits to individuals who lose their spleen to have the resident autologous cellular immune components readministered to the host. In order to address this question, we wanted to utilize Pneumovax^®^ 23, which is purely a polysaccharide vaccine, in order to observe its ability only to evoke a pure T cell-independent (TI) response from B cells [17]. Although Prevnar^®^ 13, which is a protein conjugated pneumococcal vaccine, induces a strong immune response through T helper cell-mediated humoral response. We were unsure if administering this vaccine would boost immunoglobulin (Ig) levels so high and confound the results to measure only B cell response minimizing the cascade of signals and cell interactions that may occur from T-cell activation. Future studies to evaluate the further enhancement of response by administration of protein conjugated vaccine on these harvested splenic lymphocyte populations are planned.

Our results reveal some trends that may help us reinforce the concept that there are specific lymphocyte populations that carry idiotype specific recognition of gram-negative encapsulated microorganism polysaccharide and that these autologous cells may be able to be isolated and reinfused to help individuals retain specificity in immune protection against these infections

## 2. Methods

### 2.1. Materials

PNEUMOVAX^®^23 (pneumococcal vaccine polyvalent) was manufactured at MerckVaccines.com. The pneumococcal polysaccharide vaccine (PPSV23 or PNEUMOVAX^®^23) protects against 23 serotypes of pneumococcal bacteria, including 1, 2, 3, 4, 5, 6B, 7F, 8, 9N, 9V, 10A, 11A,12F, 14, 15B, 17F, 18C, 19F, 19A, 20, 22F, 23F, and 33F. All other materials and reagents were listed as below in the procedure.

### 2.2. Animal Study

Balb/C female mice (Jackson Lab, Bar Harbor, ME, USA) weighing 20–25 g (age of 8–10 weeks) were acquired for the experiment. Specifically, 30 mice in Group A (10 for each subgroup), 30 in Group B (10 per each subgroup), and 30 in Group C (10 for each subgroup) with intravenous injection of inactivated *Streptococcus pneumoniae* Type III cells. The mice were free from pathogens and kept in filter isolation throughout the study. They were housed in an accredited animal care facility at the CHOC Children’s Hospital Research Institute and placed on a regular photoperiod with normal temperature and given laboratory chow and water, all under standard guidelines with the Institutional Animal Care and Use Committees (IACUC) approval.

### 2.3. Operative Procedure of Splenectomy

All operative procedures were approved by IACUC and detailed, as described previously [18]. Briefly, anesthetic, analgesic, tranquilizing or neuromuscular blocking agent(s) and post-anesthetic recovery: ketamine/xylazine (dose: 120/20 mg/kg) or Codeine (1 mg/kg), were applied. All operative procedures were done after the animal was induced with 2% halothane and maintained on constant flow halothane at 0.80% and 2 L via a nose cone. The abdomen was cleaned with Betadine solution and then shaved. A midline abdominal incision was made. The spleen was brought out carefully into the field of view. The spleen was removed after cauterizing attached vessels with a cautery pen and placed in a sterile solution of DPBS (pH 7.2) (Lonza, Walkersville, MD, USA) with 5% FCS (fetus bovine serum) (Atlanta Biologicals, Flowery Branch, GA, USA) and kept on ice. The sham group had the spleen mobilized and placed back into position without any other disruption. Incisional wounds were closed and secured with surgical sutures (Coated VICRYL*Plus, antibacterial with Irgacare MP* Polyglactin 910 suture, undyed braided, Ethicon Inc., Somerville, NJ, USA). The mouse was anesthetized for 5–10 min surgery under a heat lamp, so the body fluid losses were negligible; however, to be cautious, injection (SC, subcutaneous injection) of 1 cc saline was used for body fluid replacement. Mice were observed for minutes after surgery to ensure adequate analgesia and again on hourly intervals during the day of surgery for 4 h. Animal appearance, activity, and behavior (e.g., food and water intake) were closely monitored as indications of pain and discomfort. The analgesic drug (Codeine) was used if the animals appeared in pain, as evidenced by visual observation (jerking motions, lack of movement, significant weight loss, vocalizing, and lack of grooming, auditory observation of squealing) for 30 min and again in 4 h post-surgery. Codeine was given twice daily for the first few days after the surgery, if needed. Post-procedure supportive care: appropriate food and water were ensured on the cage floor after the procedure. The wound sutures were absorbed through time.

### 2.4. Isolation of Splenic Lymphocytes, FACS Analysis, and Autologous Splenocyte Reinfusion by Tail Vein Injection

Isolation of splenic lymphocytes operating on ice: the spleens removed from the mice were cut into 2 mm × 2 mm squares and placed on a cell sorter sieve made from steel wire mesh and together placed over a 60 × 15 petri dish. Using a circular grinding motion, the pieces were pressed against the screen with the plunger of a 10 mL. Syringe using PBS with 5% FCS for irrigation until mostly fibrous tissue remained on the screen.

The cell suspension was then centrifuged for 10 min at 4 °C in a Beckman rotor at 1500 rpm, and the supernatant discarded. The pellet was resuspended in 10 mL of ACK lysing buffer (NH4Cl 8.29 g/L, KHCO4 1 g/L, Na4EDTA 37.2 mg/L, pH 7.2) and incubated for five minutes at room temperature with occasional shaking. Another 10 mL of PBS/5% FCS buffer was added, and the solution was centrifuged again for 10 min at 1500 rpm, followed by the discarding of the supernatant. The resulting pellet was washed in PBS/5% FCS twice, each time centrifuging for 10 min at 1500 rpm. Finally, the pellet was resuspended in a 5 mL PBS/5% FCS buffer, placed onto 20 mL of Ficoll/Paque gradient (Pharmacia LKB, Piscataway, NJ, USA) and centrifuged for 20 min at 1500 rpm with brakes off. The interphase layer was then aspirated out and washed in PBS (pH 7.22) three times.

A cell sample stained with Trypan blue was placed on a hemocytometer and examined under the microscope for purity, viability (Group A, 98.08%; Group B, 79.38%), and counts (Group A, 28.2 million/mouse-spleen; group B, 28.32 million/mouse-spleen) for the splenic lymphocyte preparation. The resultant splenic lymphocytes were reperfused back to mice in the experiment group via tail vein injection. Fractions of the cells were used for fluorescence-activated cell sorter (FACS) analysis with fluorophore-conjugated antibodies against CD3, CD19, CD21, CD35, CD80, respectively, with corresponding isotypes as controls as described in the manufacturer’s Manual (eBioscience, Affymetrix, Inc., Santa Clara, CA, USA), as described previously [19]. Specifically, CD80-FITC (for mature B cells), CD19-PE (pan-marker for global B cells), CD3-eFluor660 (for T cells), CD21-APC (for naïve B cells) were used to determine the purity of cell types of the splenic lymphocyte preparation. As 90% of splenic lymphocytes are B cells, and the other 10%, were located in lymph nodes and circulation, both CD80 for mature B cells [20] and CD21 for naïve B cells [21] were accessed. Anti-mouse lineage with fluorophore-conjugated antibodies against CD3, CD14, CD16, CD19, CD20, CD56 (BioLegend, San Diego, CA, USA), APC anti-mouse CD21/CD35 (CR2/CR1), FITC anti-mouse CD80, Alexa Fluor 647 anti-mouse CD3, PE anti-mouse CD19, and Fc blocker for MHC I were also used. Stained cells were analyzed by following the Accuri C6 Flow Cytometer Instrument Manual (BD Biosciences, San Jose, CA, USA) as guided by Frank Zaldivar, Ph.D. and Dan Cooper, MD, at University of California Irvine Medical Center.

Balb/C mice were used as an animal model to determine primary immune response (Group A) and secondary immune response (Group B). Each group had three separate subgroups (subgroup or SG1, SG2, and SG3). SG1 (positive control) was the sham control mice with the intact spleen. SG2 (negative control) consisted of splenectomized mice without autologous splenocyte lymphocytes reinfusion (acronym of SL). SG3 (experimental subgroup) were splenectomized mice with autologous splenocyte lymphocytes reinfusion (acronym of +SL). Group A was given Pneumovax^®^23 (Pneumococcal polysaccharides, PnPS)-based vaccine PNEUMOVAX^®^23, after surgical intervention, and then weekly blood draws to evaluate antibody titers. Group B had the first vaccine after surgical intervention followed by revaccination six weeks later, and blood was drawn weekly to evaluate antibody titers.

### 2.5. Retro-Orbital Venous Plexus for the Blood Draw

Baselines (before immunization) and weekly blood draws were performed via micro-capillary connecting to retro-orbital plexus bleed of anesthetized mice (300 mL isoflurane with a cotton ball in the bottom of the jar and cover with plastic mesh) were performed for nine weeks to assess antibody response). The micro-capillary tubes were micro hematocrit capillary tubes, ammonium heparinized, green tip, 100/vial, soda lime glass (#51608, Globe Scientific Inc., Paramus, NJ, USA). The topical ophthalmic anesthetic was applied for pain relief after the procedure. Mice in all subgroups were given a second vaccine challenge (Group B). Repeat baseline and weekly blood draws were performed to assess antibody response. All mice were performed for full exsanguinations or pneumothorax on the final blood draw; mice were given ketamine and xylazine overdosage to release pain, then cervical dislocation was performed, and breath and heartbeat were used as an indication of death. Medication for pain was administered on routine postoperative and post-procedural monitoring if the pain was detected. Discomfort from blood draws was minimal (topical anti-pain and antibiotic ointment were applied), and the animals were monitored similar to when they had their operative procedure. They were given ketamine/xylazine (see 8.c, below) if they exhibited symptoms of pain. SC injection of Anesthetic Ketamine (120 mg/kg) or SC injection of analgesia/ sedation xylazine (20 mg/kg) was administrated every 4 h for relief of postoperative pain, later as needed. Sera were separated by micro-capillary centrifuge (Model MB, International Equipment Company, Needham, MA, USA) and stored at −20 °C for antibody testing.

### 2.6. ELISA for Antibody Concentration Measurements

Enzyme-linked immunosorbent assay (ELISA) was performed to determine IgM and IgG antibody titers on the specimen. Pneumococcal polysaccharides (PnPS)-based vaccine PNEUMOVAX^®^23 (Pneumococcal Vaccine Polyvalent 23 serotypes of serotypes 1, 2, 3, 4, 5, 6B, 7F, 8, 9N, 9V, 10A, 11A,12F, 14, 15B, 17F, 18C, 19F, 19A, 20, 22F, 23F, and 33F) were coupled to protein for adsorption, in accordance with the procedure set forth by Gray [22], with the modifications of components [23], of ELISA format for serum samples derived from infants vaccinated with conjugate vaccines [24], and of verified patient serum post-PPSV23 vaccination [25]. Briefly, three test tubes A, B, C were prepared such that tube A had 0.5 mL of 0.05 N NaOH with 0.001% phenolphthalein; tube B had 1 µg of cyanuric chloride crystals, and tube C had 0.1 mL of 0.2% poly-L-lysine (MW 54,000, Sigma Chemicals, St. Louis, MO, USA). A polysaccharide solution of l00 μL (2.5 μg/mL) of PPSV23 was alkalinized for 10 s by swirling in tube A. Activation was then accomplished by pouring the contents of tube A into tube B, and swirled the contents for 10 s, at which point the solution turned colorless. The test tube contents were then coupled to poly-L-lysine in tube C and refrigerated at 4 °C for 2 h. The coupled polysaccharide was diluted in a 1:4 ratio in PBS (pH 7.2) and eventually used for adsorption onto ELISA plates to test for antibody titers after serial dilutions were titrated in order to determine the optimal linear range of standard curves suitable for the ELISA plate reader. Antibody titers against *Strep. pneumoniae* polysaccharide was quantified by the ELISA system: Mouse IgG (or IgM, accordingly) total Ready-SET-Go 10x96 tests (eBioscience, Affymetrix, Inc., Santa Clara, CA, USA). ELISA plates were coated with 50 μL of pneumococcal polysaccharide vaccine coupled to poly-L-lysine. Plates were then incubated at 37 °C for 2 h and then washed three times in PBS with 0.5% Tween. All free sites on the plate were blocked using PBS-Tween (0.5%)-blocking buffer (1% bovine albumins) and incubated at 37 °C for 2 h with rocking (3-D Rotator, Lab-Line Instruments, Inc., Beaumont, TX, USA. The plates were rinsed again 3× in PBS-Tween (0.5%). Subsequently, the mouse antiserum went through a series of dilutions on the plates and incubated overnight at 4 °C. All wells were again washed three times in PBS-Tween (0.05%). Next, 50 μL of a 1/100 dilution of goat anti-mouse Ig (or IgM, accordingly) antisera linked to alkaline phosphatase was added to each well and incubated at 37 °C for 1 h. All wells were rinsed three times in PBS-Tween (0.05%). Finally, 50 μL of *p*-nitrophenol 1 phosphate (1 mg/mL) (Sigma Chemicals) in diethanolamine buffer (pH 9.6) was added to each well and incubated for 30 min before reading on an ELISA reader—Vmax^®^ Kinetic ELISA Microplate Reader (Molecular Devices LLC., Sunnyvale, CA, USA). The concentration of antibody was determined by following the methods as described previously [26] for enzyme immunoassay units [27], with logarithmically transformed to normalize [27].

### 2.7. Preparation and Inoculation with Nonviable Streptococcus pneumoniae Cells

*Streptococcus pneumoniae* Type III cells were purchased from ATCC (supplier, Bethesda, MD, USA. These cells were inoculated into tryptic soy broth (Difco Labs, Detroit, MI, USA), previously adjusted to pH 7.7, and grown for 4–6 h at 37 °C. Formaldehyde was added to a concentration of 0.1%, and the cell suspension was stored at 4 °C after being washed three times in PBS (pH 7.2). Before use, the cultured cells were washed 3× in sterile PBS and centrifuged into a pellet. They were also test plated onto chocolate agar to ensure non-viability and tested for the presence of a capsule with an India ink stain and Quellung positive with Pneumococcus type III specific antisera (Difco). The Group C mice were then immunized with 1 × 10^3^ of the prepared nonviable *Streptococcus pneumoniae* cells intravenously, using McFarland nephelometry for quantification.

### 2.8. Statistical Methods

Statistical analysis was performed by Dan M. Granoff, M.D., FPIDS, Clorox Endowed Chair and Director, Center for Immunobiology and Vaccine Development, UCSF Benioff Children’s Hospital Oakland Research Institute, 5700 Martin Luther King, Jr. Way, Oakland, CA, USA. Specifically, statistical analysis software (GraphPad Prism 8.2) (GraphPad Software, San Diego, CA USA) was used. Raw *p*-values were obtained using the two-tailed *t*-test of the mean without strata adjustment. *p*-values were also adjusted with the Step-Down Bonferroni Adjustments method. Both raw *p*-values and adjusted *p*-values are reported. The adjusted *p*-values were somewhat larger than the raw *p*-values, which reflect a correction for conducting multiple tests. The *p*-value was obtained using a two-tailed *t*-test of the mean. Differences were considered statistically significant for *p* < 0.05.

## 3. Results

**Figure 1:**
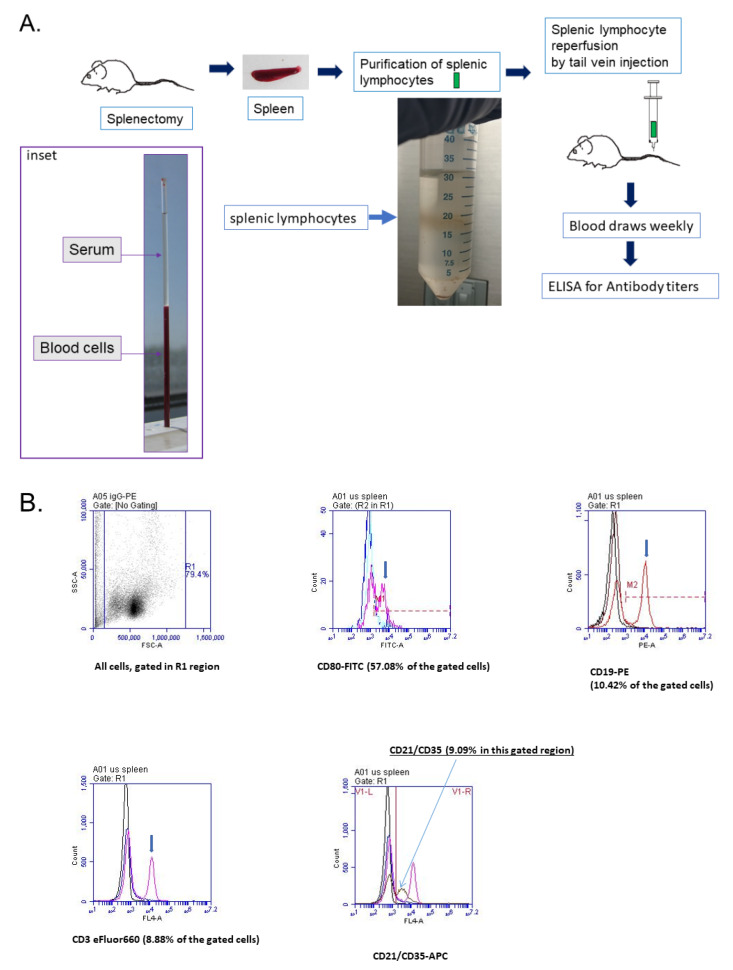
Summary of the procedure for restoring the innate humoral antibody-mediated immunity responsive to *Pneumococcal* polyvalent 23 serotypes (Pneumovax^®^23) through autologous splenocyte reinfusion upon splenectomy mice. (**A**) Flow chart of the procedure—both IgM immune response after initial vaccination (Group A) and IgG immune response following revaccination (Group B). Each group had three separate subgroups (1, 2, and 3). Subgroup 1 (positive control subgroup) were the sham control mice, subgroup 2 (negative control subgroup) consisted of splenectomized mice (−SL), and subgroup 3 (experimental subgroup) consisted of splenectomized mice with autologous splenic lymphocytes (+SL) reinfusion. Inset: micro hematocrit capillary tubes were used to separate serum from blood cells, derived from blood draw via retro-orbital venous plexus. (**B**) Representatives of FACS analysis of splenic lymphocytes. Fractions of the cells (#062513) were used for FACS analysis with fluorophore-conjugated antibodies against CD3, CD19, CD21, CD35, CD80, respectively, with corresponding isotypes as controls as described in the manufacturer’s Manuel (eBioscience, Affymetrix, Inc., Santa Clara, CA, USA), as described in Methods. Specifically, CD80-FITC (for mature B cells), CD19-PE (pan-marker for global B cells), CD3-eFluor660 (for T cells), CD21-APC (for naïve B cells) were used to determine the purity of cell types of the splenic lymphocyte preparation. As the majority of splenic lymphocytes are B cells and the other B cells are located in lymph nodes and circulation, both CD80 for mature B cells and CD21 for naïve B cells were accessed (Note: Gate R1, 79.4% of the cells were used for analysis regions that illustrated as the analysis histograms).

Clinical outcomes of surgical splenectomy depend on many variables, including the training and expertise of the surgeon. The proper surgical technique can limit time under anesthesia, blood, and fluid loss, and can lower infectious risk, which all directly impact the consistency in measuring host immune responses, given that stress and inflammation pose significant variables in such studies [18]. As shown in Figure 1A, we designed and executed the procedure to obtain mouse spleens and isolated the splenic lymphocyte fraction that we would use for restoring the innate humoral antibody-mediated immune response to Pneumococcal Polyvalent 23 serotypes (PNEUMOVAX^®^23, PPSV23) through autologous splenocyte reinfusion on splenectomized mice. The isolated splenic lymphocytes consisted of T lymphocytes and B lymphocytes, as detected by FACS analyses (Figure 1B). Next, we wanted to know if the isolated splenic lymphocytes could restore the innate humoral antibody-mediated immune response to PPSV23 by measuring the IgM response after initial vaccination and the IgG response after revaccination, as well as against whole *Streptococcus pneumoniae* type III cells. We collected low volume blood samples at timed intervals after exposure to the vaccine in order to perform antibody titers using the ELISA technique. All three groups of animal studies were shown in Table 1 in detail.

**Figure 2:**
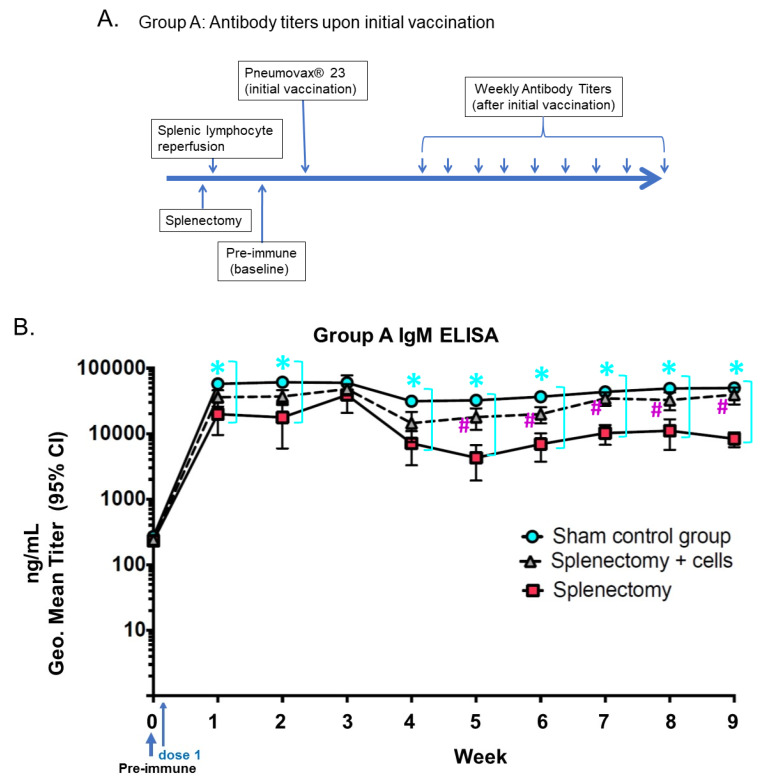
Group A mice-humoral antibody-mediated immune response to initial PPSV23 vaccination (**A**) immediately following splenectomy, as measured by IgM ELISA (**B**). Y-axis: Absorbance IgM ng/mL, Geo. Mean (i.e., geometric mean) antibody titer (95% CI). *X*-axis: time interval in a week post PPSV23 vaccination. Antibody IgM titers of Balb/C mice with the initial vaccination and tested against polysaccharides conjugated to ELISA plates. Antibody IgM titers of mice immunized with initial vaccination of PPSV23 polyvalent pneumococcal vaccine, with baseline (i.e., pre-immune) antibody titers not exposed to any pneumococcal antigen. Briefly, humoral immune response (Group A). Group A had three separate subgroups (1, 2, and 3). Subgroup 1 (positive control) was the sham control of 10 mice, subgroup 2 (negative control, splenectomy alone) consisted of 10 splenectomized mice (−SL, i.e., without reinfusion of autologous Splenic Lymphocytes), and subgroup 3 (experimental subgroup) consisted of 10 splenectomized mice (+SL, i.e., with reinfusion of autologous Splenic Lymphocytes). The control animals undergoing a sham laparotomy with spleen intact had the best antibody responses. Splenectomized mice (−SL) had the lowest antibody responses. The subgroup of splenectomized mice (+SL) had an intermediate antibody response. This elevated response in the experimental subgroup (+SL) mice had an immune response as detected by elevated IgM titers in Group A. (* signals the statistically significant, *p* < 0.001, sky-blue circles (sham control group) compared with red squares (−SL subgroup). # for statistically significant, *p* < 0.001, grey triangles (+SL) compared with red squares (splenectomy)) (Geo. Mean ± S.D. concentrations (ng/mL titers) and 95% confidence intervals as measured by ELISA with Log_10_ scale) (“Geo.” stands for geometric mean).

As shown in Figure 2, Group A (Balb/C mice) was used to determine immune response after initial vaccination by the measurement of antibody IgM concentrations. This figure plots absorbance on the *y*-axis versus time on the *x*-axis. The absorbance directly relates to antibody titer in mouse serum. The splenectomized subgroup where splenic lymphocytes were reinfused, were compared to the antibody titers of the splenectomized subgroup with no reinfusion of splenic lymphocytes and the sham group that still had the spleen intact. The plot shows the antibody titers to subcutaneous vaccination with Pneumovax^®^23 (PPSV-23) polysaccharide antigens over time. It is noted that peak titers were reached within one-week post-immunization for the sham subgroup and stayed the same level over nine weeks; however, subgroup mice (−SL), as well as the subgroup mice (+SL), peaked in week three, and then the IgM level dropped. As expected, non-splenectomized animals had higher antibody titers to PPSV23 vaccination compared to splenectomized mice. The splenectomized subgroup with splenic lymphocyte reinfusion had an elevated immune response at one week compared with the subgroup without splenic lymphocytes. We observed that the control animals undergoing a sham laparotomy with spleen intact had the best antibody responses to vaccine challenge. Splenectomized mice (−SL) had the lowest antibody responses. The subgroup of splenectomized mice (+SL) had an intermediate antibody response. This elevated response in the experimental subgroup (+SL) mice had a response as detected by elevated IgM titers in Group A.

Non-splenectomized animals demonstrated higher antibody titers in reaction to the PPSV23 vaccination compared to the splenectomized mice (−SL, +SL). The splenectomized subgroup with splenic lymphocyte reinfusion (+SL), had an elevated immune response at one week compared with the subgroup without splenic lymphocytes (−SL). The reinfused splenic lymphocytes provided to the splenectomized subgroup strengthened the humoral immune response, as the spleen serves as a repository for specific subsets of lymphocytes that may be reactive to antigens such as polysaccharide antigens on encapsulated gram-negative organisms. We found that antibody response declined in all subgroups after week three; response in +SL subgroup as compared to −SL subgroup was significantly higher beginning in Week five (*p* < 0.001); response in +SL subgroup was near comparable to response in non-splenectomized subgroup mice.

**Figure 3:**
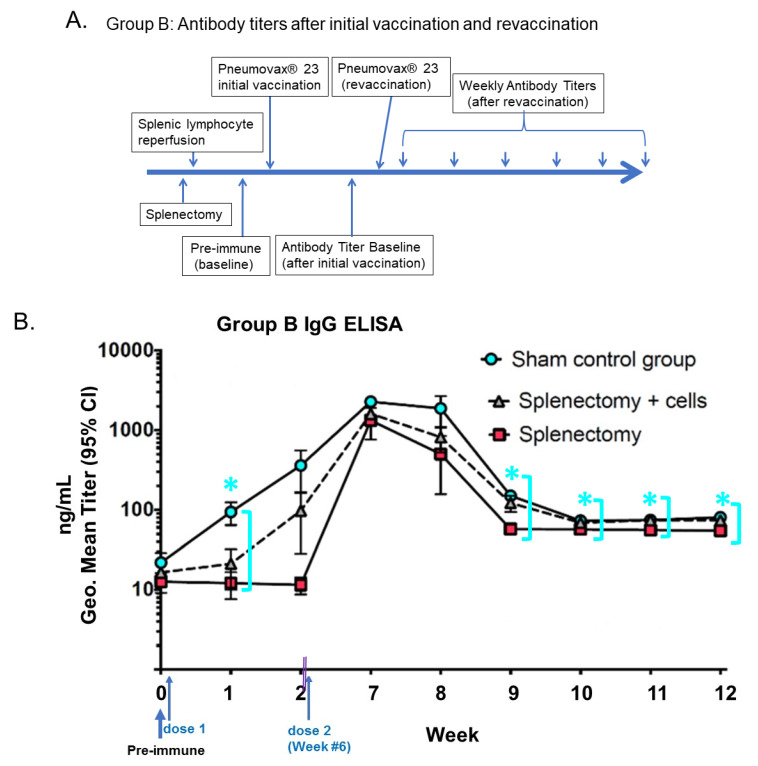
Group B mice; a humoral antibody-mediated immune response to PPSV23 vaccination (**A**) immediately following splenectomy, with revaccination at 6 weeks post-splenectomy by IgG ELISA (**B**). *Y*-axis: Absorbance IgG ng/mL, Geo. Mean (i.e., geometric mean) antibody titer (95% CI). *X*-axis: time interval in weeks post PPSV23 vaccination. Antibody IgG titers of Balb/C mice with the repeat vaccination (6 weeks) and tested against polysaccharides conjugated to ELISA plates. Baseline (i.e., pre-immune) antibody titers of mice were not exposed to any pneumococcal antigen. This elevated response in the experimental group (+SL, i.e., with reinfusion of autologous Splenic Lymphocytes) mice had an immune response as detected by elevated IgM titers in Group A (Figure 2) and as IgG titers in Group B (Figure 3). There was also the mortality of 5% in Group B splenectomized mice (−SL, i.e., without reinfusion of autologous Splenic Lymphocytes) and 40% in Group B splenectomized mice (+SL) near the end of the study period. The Group B splenectomized mice (+SL) had equivalent titers over the long term when monitored out to six weeks as opposed to a low and falling titer observed in the Group B splenectomized mice (without SL). (* signals the statistically significant, *p* < 0.001, sham control subgroup being compared with −SL subgroup for weeks 10, 11, 12), as sky-blue circles compared with red squares (splenectomy), as shown with mean ± S.D. concentrations (ng/mL titers) and 95% confidence intervals as measured by ELISA with Log_10_ scale). Doubled-purple-lines defined a time gap from 2 weeks to the repeat vaccination at 6 weeks. (“Geo.”: it stands for geometric mean.).

As shown in Figure 3, Balb/C mice were used to determine the immune response (Group B). Group B had the first vaccine after surgical intervention and was given a second vaccine (PPSV23) six weeks later and then had blood drawn every week, subsequently, to ascertain antibody titers in the blood post-revaccination up to 12 weeks, with two blood draws for assessment of the immune response of the first-vaccine as a comparison. ELISA panels were run in various serum dilutions to determine the immune response to repeat vaccination through IgG antibody concentration (Figure 3), as compared to the immune response to initial vaccination by the measurement of antibody IgM (Figure 2). Statistical analysis performed showed that secondary response as detected by elevated IgG titers in Group B across all the subgroups (1, 2, and 3) and the control animals (subgroup 1) undergoing a sham laparotomy with spleen intact had the best antibody responses. In contrast, splenectomized mice without splenic lymphocytes reperfusion (−SL) had the lowest antibody responses. The subgroup of splenectomized mice with splenic lymphocytes reperfusion (+SL) had an intermediate antibody response. There was also a significant difference in mortality rates across the three subgroups, with 5% mortality of subgroup splenectomized mice (+SL) compared to 40% mortality in subgroup splenectomized mice (−SL) near the end of the study period, indicating that autologous splenocyte reinfusion upon splenectomized mice might have survival benefits not isolated to only infection risks. The subgroup B splenectomized mice (+SL) had equivalent titers over the long term when monitored to six weeks post-revaccination, as opposed to a low and falling titer observed in the subgroup, splenectomized mice (−SL). All of the graphs remained stable (flat) in Weeks 9–12.

**Figure 4:**
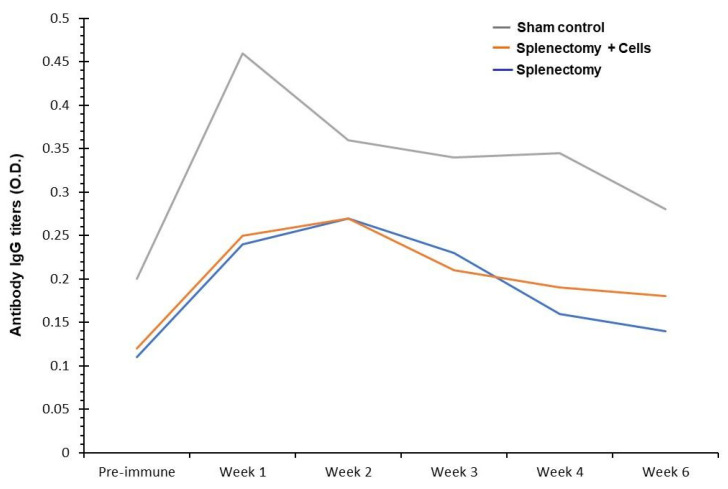
Group C mice: Antibody IgG titers were upregulated in mice upon challenges by intravenous injection of inactivated *Streptococcus pneumoniae* Type III cells as humoral antibody-mediated immune response. Whole *Streptococcus pneumoniae* Type III cells were inactivated by 0.1% formalin treatment and washed 5x in PBS (pH 7.2) before intravenous injection. *Y*-axis: Optical density measurement units reflected antibody IgG titers on the ELISA plate reader. *X*-axis: time interval in a week. The grey line: sham mice possess their spleen and its constituent lymphocytes. The orange line: splenectomized mice (+SL) that did not possess a spleen, but had their splenic lymphocytes reinfused via tail vein injection. The blue line: splenectomized mice (−SL) that did not possess a spleen and did not have any splenic lymphocytes reinfused.

Next, we wanted to determine if *Streptococcus pneumoniae* Type III bacteria activate such a response as triggered by the PPSV23 vaccine. Figure 4 shows that when Group C mice were given killed (inactivated) *Streptococcus pneumoniae* Type III bacteria intravenously, the sham subgroup displays a peak antibody response in one week, as seen in the subgroup with polysaccharide challenge. The lines denote the same subgroups used in the previous Figure 2 and Figure 3. Here, the non-splenectomized control subgroup exhibits an elevated antibody response in one week. Both of the splenectomized subgroups (with and without reinfused splenic lymphocytes) show a lower, yet somewhat elevated, response peaking approximately two weeks after administration of killed *Streptococcus pneumoniae* cells. However, neither of the splenectomized subgroups with and without splenic lymphocyte reinfusion showed an enhanced response to a killed *Streptococcus pneumoniae* challenge (Figure 4). Based on the graph in Figure 4, it looks like the +SL response was similar to the −SL response; some divergence is seen in weeks 4–6 with a greater immune response for +SL–, but was no statistically significant difference, given the number of 10 mice in each subgroup.

## 4. Discussion

The first splenectomy done by Riegner in 1892 led to a whole era of “curative splenectomy [28],” however, King and Shumacker reported the syndrome of overwhelming post-splenectomy infection (OPSI) in 1952, which dampened surgical enthusiasm for splenectomies [29,30]. Since then, the risk/benefit ratios of performing splenectomies have had to be reconsidered. Removal of the spleen, whether indicated by trauma, malignancy, or blood dyscrasias, appears to increase the risk of post-splenectomy sepsis, primarily if performed in childhood [31]. It can be rationalized that if the spleen serves as a filter for intravascular bacterial contaminants, a possible way of decreasing the risk of post-splenectomy sepsis would be to save the spleen or to autotransplant sections that can be salvaged. Indeed, researchers have found that preservation of the autologous spleen through autotransplant of devascularized splenic tissue into the omentum is associated with the oligoclonality, polyclonality, significant increase of IgM, and increase of C3 in splenectomized patients [32]. After splenectomy, a patient typically suffers a reduced immunity to certain gram-negative bacteria resulting from a loss of specific immune functionality provided by the spleen. This may be related to the functional structure of the spleen or directly associated with cellular subpopulations.

Spleen loss may lead not only to a reduced cell-mediated immune response (through T-lymphocytes and other macrophages) but also to a reduced humoral immune response due to a loss of B-lymphocytes, antibodies produced by these B-lymphocytes, and the loss of spleen structure functioning in antigen presentation and removal. Thus, splenectomy may lead to reduced immune response, not only because of the loss of splenic architecture in mediating the immune response, but also due to the loss of the significant population of cells and other constituents, including lymphocytes. These lymphocytes may include, but are not limited to, B cells (including plasma B cells, memory B cells, and follicular B cells), T cells (including cytotoxic T cells, memory T cells, helper T cells, natural killer T cells, suppressor T cells, and gamma delta T cells), natural killer cells, and progenitor lymphopoietic stem cells. Splenic lymphocytes and the immune-boosting constituents may be isolated from the salvaged spleen of a patient undergoing splenectomy and reintroduced to help restore potentially functional and specific components of their immune system for future protection from infection by gram-negative encapsulated organisms. Interestingly, even though this study is focused on potential immune enhancement through this process, we find in reviewing the literature that patients seemed more at risk of cardiac death after splenectomy perhaps due to loss of monocyte reservoirs crucial in tissue repair as well as increased associations with type II diabetes and childhood obesity [33] and type I diabetes [34]. We also found subsets of patients such as those with the autoimmune lymphoproliferative syndrome (ALPS) who do much worse after splenectomy. We also mentioned, previously, the need for partial splenectomy, not only to preserve immune function, but to diminish the risk of developing pulmonary hypertension over time. This occurs due to the significant filtering burden carried out by the spleen, which gets shifted to the pulmonary vasculature in asplenic patients leading to increases in pulmonary hypertension [1].

We started this study by asking if reinfusion of autologous splenic lymphocytes (+SL) would result in improved antibody response to PNEUMOVAX^®^23 in splenectomized mice. We observed that the control animals undergoing a sham laparotomy with spleen intact had the best antibody responses. Splenectomized mice (−SL) consistently had the lowest antibody responses of all the Groups A, B, and C. The subgroup of splenectomized mice (+SL) had an intermediate antibody response. This elevated response in the +SL subgroup mice was detected after initial vaccination (measured by IgM titers) in Group A and after repeat vaccination (measured with IgG titers) in Group B. There was also the mortality of 5% in Group B splenectomized mice (+SL) at week 10 of the study period and 40% in Group B splenectomized mice (−SL) at week 7 of the study period. The Group B splenectomized mice (+SL) had equivalent titers over the long term when monitored out to six weeks as opposed to a low and falling titer observed in the Group B splenectomized mice (−SL). In the “sham control mice,” no “secondary IgG response” is found, but a progressive increase of IgG titer following the first and second immunization. We also notice that the levels of IgG after revaccination are much lower in concentration than the levels of IgM after the initial vaccination. It is unclear if these levels are clinically effective since the sham group, which still has a completely intact and immunocompetent system, also showed similar lower IgG concentrations or if these lower levels resulted from “hyporesponsiveness” to repeat polysaccharide vaccination as has been shown in other studies [35]. In contrast, a report of enrolling participants (*n* = 143, mean age 76 years) concluded that “Antibody IgG persistence ten years after first and second doses of 23-valent pneumococcal polysaccharide vaccine (PN23), and immunogenicity and safety of second and third doses in older adults” and “Immunogenicity is preserved after multiple PN23 doses” (without evidence of a lower than expected immune response (i.e., without hyporesponsiveness) [25]. Another report indicated that “Revaccination with a 23-valent pneumococcal polysaccharide vaccine induces elevated and persistent functional antibody IgG responses in adults aged equal to 65 years or older, in 3–5 years after receiving a first PN23 vaccination (*n* = 60)” [27].

The Group C results (Figure 4) indicate the possibility of a more involved mechanism of response towards T-dependent antigens (e.g., bacteria, virus-infected cells, tumor cells) not easily rectified by a reinfusion of lymphocytes. Increased immunity may involve both the structure of the spleen in conjunction with the reticuloendothelial system or a lack of pre-existing antibodies that may be used to opsonize the killed cells. Studies have shown that the spleen is responsible for the clearance of T-dependent antigens such as bacteria compared to the liver and other organs [36]. As such, post-splenectomy cellular immunity may be improved by immunization before the splenectomy, as antibodies against T-dependent antigens would then permit such antigens to be cleared without the spleen. This same model also needs to be evaluated after initial vaccination with Prevnar^®^, which is a T dependent protein conjugated vaccine for *Streptococcus pneumoniae*, which we hope to do in future studies.

Our finding corroborates previous experimental and clinical studies demonstrating an impaired immunologic response and increased susceptibility to infection in asplenic individuals. Pneumococcal vaccines should be given before non-emergency splenectomy. Alternatives to splenectomy with conservative management should be considered for patients with splenic trauma when possible [15]. In particular, strong consideration should be given to autologous splenic lymphocytes as vital and simple to readminister to patients undergoing splenectomy. This may serve to reduce the likelihood of a post-splenectomy infection and possibly other less studied complications

## 5. Conclusions

Improvements in humoral antibody-mediated immune response may be achieved against encapsulated gram-negative organisms by isolating splenic lymphocyte fractions and readministering them to patients undergoing splenectomy. More detailed studies will need to be performed to understand further the contributions achieved by challenging and priming the immune system with Prevnar^®^ protein conjugated vaccine in comparison to the current study with PNEUMOVAX^®^23, which is strictly a polysaccharide vaccine. In addition, studies utilizing selective deletion of T and B cell components can add additional support to the necessary components of this observed response. It will also help to delineate specific subpopulations of cells within these isolated reinfused cellular fractions.

## Figures and Tables

**Table 1:** All the different types of subgroups/groups and related treatment.

Group of 30 Mice	10 Mice for Each Subgroup	Surgery (Splenectomy)	Reinfusion of Autologous Splenic Lymphocytes (SL)	Primary Vaccination	Boost Vaccination	Intravenous Injection of Inactivated *Streptococcus pneumoniae*	Blood Draw for Antibody Titer
**Group A**	A1 (Sham positive control)	-	N/A	+			+
	A2 (Negative control)	+	-	+			+
	A3 (Experimental)	+	+	+			+
**Group B**	B1 (Sham positive control)	-	N/A	+	+		+
	B2 (Negative control)	+	-	+	+		+
	B3 (Experimental)	+	+	+	+		+
**Group C**	C1 (Sham positive control)	-	N/A	+		+	+
	C2 (Negative control)	+	-	+		+	+
	C3 (Experimental)	+	+	+		+	+

Note: refer to the body of text for details.
